# The Impact of Modern Bone Markers in Multiple Myeloma: Prospective Analyses Pre and Post-First Line Treatment

**DOI:** 10.3390/cimb46090552

**Published:** 2024-08-24

**Authors:** Vlad Stefan Pop, Mihaela Iancu, Adrian Bogdan Țigu, Anda Adam, Gheorghe Tomoaia, Anca Daniela Farcas, Anca Simona Bojan, Andrada Parvu

**Affiliations:** 1 Hematology Department, “Iuliu Hatieganu” University of Medicine and Pharmacy, 400124 Cluj-Napoca, Romania; simona.bojan@umfcluj.ro (A.S.B.); parvuandrada@hotmail.com (A.P.); 2 Oncology-Hematology Department, Emergency Hospital, 2 Ravensburg Str., 440192 Satu Mare, Romania; 3 Department of Medical Informatics and Biostatistics, “Iuliu Hațieganu” University of Medicine and Pharmacy, 400012 Cluj-Napoca, Romania; 4 Department of Translational Medicine, Institute of Medical Research and Life Sciences—MEDFUTURE, “Iuliu Hațieganu” University of Medicine and Pharmacy, 400337 Cluj-Napoca, Romania; 5 General Pharmacy, Clinic Emergency Hospital Sibiu, 2-4 Corneliu Coposu Blvd, 550245 Sibiu, Romania; adam_anda93@yahoo.com; 6 Orthopedics and Traumatology Department, “Iuliu Hatieganu” University of Medicine and Pharmacy, 400132 Cluj-Napoca, Romania; tomoaia2000@yahoo.com; 7 Internal Medicine Department, “Iuliu Hatieganu” University of Medicine and Pharmacy, 400006 Cluj-Napoca, Romania; ancafarcas@yahoo.com; 8 Cardiology Department, Emergency County Clinic Hospital, 400006 Cluj-Napoca, Romania; 9 Hematology Department, “Prof. Dr. Ioan Chiricuta” Oncological Insitute, 400124 Cluj-Napoca, Romania

**Keywords:** multiple myeloma, modern bone markers, IL-6, free soluble RANKL, IFN-beta

## Abstract

Multiple myeloma, the disease characterized by the malignant proliferation of plasma cells that invades the bone marrow, produces osteolytic lesions and secretes monoclonal proteins. Several biomarkers have been shown to represent important tools in the pathogenesis of myeloma and offer insights into bone degradation and formation. The objectives of this current study were to assess the associations of modern biomarkers (TNF-α: tumor necrosis factor; IFN: Interferon; FreeRANKL: Free Receptor Activator for Nuclear Factor kappa B Ligand; RANKL: Receptor Activator for Nuclear Factor kappa B Ligand, Beta crosslaps, IL-6: Interleukin 6) with osteolytic lesions status after first-line treatment and to evaluate the correlations between modern and classical biomarkers (LDH: Lactate Dehydrogenase; VSH: Erythrocyte Sedimentation Rate; Hgb: Hemoglobin, Calcium, Albumin, B2microglobulin) stratified by osteolytic lesions status. A total of 35 patients diagnosed with multiple myeloma divided into two groups according to the osteolytic bone lesions, were studied: (1) unchanged status of osteolytic lesions and (2) changed status of osteolytic lesions. After fist-line treatment, we found a significant difference in Albumin (*p* = 0.0029) and Calcium levels (*p* = 0.0304), patients with a changed status in osteolytic lesions having higher values of Albumin and Calcium compared to those without changes in status of osteolytic lesions. After first-line treatment, decreased IL-6 values were significantly correlated with elevated values of Albumin (ρ = −0.96, *p* = 0.0005) in the patients with changed status of osteolytic lesions. Post-treatment values of IFN showed a significant positive correlation with Hemoglobin (ρ = 0.47, *p* = 0.0124), IL-6 (ρ = 0.55, *p* = 0.0026) and TNF-alpha values (ρ = 0.54, *p* = 0.0029). The results obtained from patients with unmodified lytic lesions identified a significant correlation between the biomarkers IL-6, Free RANKL, and IFN-beta with the classical marker LDH. This association highlights the involvement of these markers in promoting bone destruction and the development of osteolytic lesions.

## 1. Introduction

Multiple myeloma (MM) is an incurable neoplasm considered a hematological cancer characterized by clonal expansion of malignant plasma cells accumulating in the marrow, leading to cytopenia, hypercalcemia, renal dysfunction, hypogammaglobulinemia, and osteolytic bone disease. The malignant cells produce monoclonal antibodies, typically immunoglobulin G or immunoglobulin A, and proliferate and mature in the bone marrow [1,2].

The incidence accounts for 10% of all hematological malignancies with a median age at diagnosis of 72 years [3].

The updated diagnostic criteria for myeloma represent a change in the approach to the disease with a considerable impact on its management. The diagnosis of multiple myeloma required the presence of a terminal organ lesion known as CRAB criteria, hypercalcemia, renal dysfunction, anemia, and bone damage. The IMWG (International Myeloma Working Group) criteria include both the CRAB criteria and three defining myeloma events (MDE): clonal bone marrow plasma cells of either 60% or more, the serum-free light chain of 100 or higher, and at least one focal lesion on MRI studies. The presence of at least one of these markers is considered sufficient to establish a diagnosis of multiple myeloma [4,5,6].

In myeloma, bone disease is a serious problem that often causes pain and pathological bone fractures at different locations. For example, vertebral osteolysis can cause pathological fractures and even spinal collapse with spinal cord compression and neurological damage. Given the multitude of clinical manifestations in MM, such as renal failure, hyperviscosity, and anemia, this bone disease has the greatest impact on quality of life [3,5,6,7].

Several studies have explored the levels of serum cytokines in patients with MM, discovering a correlation between high levels of certain cytokines and the presence of aggressive or symptomatic disease [8]. Additionally, research has identified prognostic factors such as genetic or epigenetic abnormalities and immune system anomalies that may help predict the course of myeloma in patients. Despite advancements in therapies that have enhanced the prognosis for MM patients, the disease remains incurable. It is crucial to note that not all cytokines are involved in the pathogenesis, progression, or prognosis of MM. The most important cytokines include IL-6, RANKL, TNF-α, and β-crosslaps (β-CTx) [7,8,9,10].

Cytokines are essential in the pathogenesis and advancement of multiple myeloma, particularly in relation to bone diseases. Interleukin-6 functions as a growth factor for multiple myeloma cells, playing a significant role in their resistance to apoptosis and promoting the formation and activity of osteoclasts. Moreover, tumor necrosis factor-alpha is instrumental in osteoclastogenesis, which leads to bone resorption and is linked to the development of bone lesions in multiple myeloma patients [11]. Additionally, β-CTx and RANKL serve as markers for bone resorption, and their interactions with other biological markers can indicate disease progression and forecast outcomes in bone metabolism [12,13].

This current study assessed the following objectives: (i) to test the associations of modern biomarkers (measured at baseline and after first-line treatment) with osteolytic lesion status after first-line treatment, (ii) to evaluate the cross-sectional correlations between modern and classical biomarkers stratified by osteolytic lesions status, and (iii) to evaluate the longitudinal correlations between changes in modern and classical biomarkers stratified by osteolytic lesions status.

## 2. Materials and Methods

### 2.1. Sample Collection and Patient Inclusion

This research represents a prospective study analysis involving 35 participants, conducted from 2019 to 2021.

This study adhered to the principles outlined in the Helsinki Declaration and received approval from the Ethics Committee of “Iuliu Hatieganu” University of Medicine and Pharmacy in Cluj-Napoca, no. 26/07.02.2022, as well as from the Ethics Committee of the Oncological Institute “Prof. Dr. Ion Chiricuta” in Cluj-Napoca, no. 89/16.03.2021.

Peripheral blood was collected on clot activator tubes and the serum was isolated after a centrifugation step at 3000× *g*, at room temperature, for 15 min. The serum was stored at −80 °C until further processing.

To be included in this study, patients were required to satisfy the diagnostic criteria for multiple myeloma, which were clonal bone marrow plasma cells >10% or biopsy-proven bony or extramedullary plasmacytoma and any one or more of the following CRAB features and myeloma-defining events: (1) Evidence of end organ damage;—hypercalcemia: serum Calcium >0.25 mmol/L (>1 mg/dL) higher than the upper limit of normal or >2.75 mmol/L (>11 mg/dL); renal insufficiency: creatinine clearance <40 mL/min or serum creatinine >177 mol/L (>2 mg/dL); anemia: Hemoglobin value of >20 g/L below the lowest limit of normal or a Hemoglobin value <10 mg/dL; and bone lesions: one or more osteolytic lesions on skeletal radiography, CT, or PET/CT. (2) Any one or more of the following biomarkers of malignancy or myeloma-defining events: 60% or greater clonal plasma cells on bone marrow examination; serum involved/uninvolved free light chain ratio of 100 or greater; provided the absolute level of the involved light chain is at least 100 mg/L, more than one focal lesion on MRI, minimum 5 mm or greater in size [13].

Those who did not meet these criteria, along with patients who opted not to sign the informed consent, were excluded from participation.

In the first month subsequent to the administration of the final first-line treatment course, the patients were assessed using imaging techniques. Our study employed Whole Body Low Dose CT (WBLDCT) and MRI as the imaging modalities to evaluate the status of osteolytic lesions.

Bisphosphonates were administered as a preventive strategy for the management of bone disease. This intervention led to a decrease in skeletal-related events and provided relief from bone pain.

### 2.2. Immunoenzymatically Testing (ELISA)

The free soluble RANKL (the receptor activator of nuclear factor kappa B ligand), human RANKL, IFN-beta, TNF-alpha, and IL-6 were analyzed using ELISA Assay from the collected serum samples, as follows:

***Free soluble RANKL*** was quantified using an ELISA assay kit from Biomedica Medizinprodukte GmbH (Biomedica Medizinprodukte GmbH, Wien, Austria—cat. no. BI-20462) using a standard curve with a range between 0 pg/mL and 2 pg/mL. For each sample, 150 μL of serum was plated according to the manufacturer’s protocol followed by 2 h incubation at room temperature. After the incubation, the plate was washed and 200 μL of biotinylated antibody anti-RANKL was added to each well and incubated overnight at 2–8 °C. Then, the plate was washed and 200 μL of conjugate was added to each well followed by one hour of incubation at room temperature. The conjugate was washed and 200 μL of substrate was added and incubated for 30 min. Then, the stop solution was added (50 μL) and the plate absorption was analyzed using a TECAN SAPRK 10M spectrophotometer (TECAN, Austria GmbH, Grodig, Austria) at 450 nm.

***Human RANKL*** was quantified using an ELISA assay kit from Elabscience (Elabscience Biotechnology Inc., Huston, TX, USA—E-EL-H5813) using a standard curve range between 15.63 and 1000 pg/mL. An amount of 100 μL of serum was used for the determination of each sample, and further steps were performed according to the manufacturer’s protocol. At the end, stop solution was added (50 μL) and the plate absorption was analyzed by TECAN SAPRK 10M spectrophotometer (TECAN, Austria GmbH, Grodig, Austria) at 450 nm.

***IL-6*** *(cat nr. DY206-05)*, ***TNF-alpha*** *(cat nr. DY210-05)*, and ***IFN-beta**  (DY814-05)* were quantified using three duo-set ELISA assay kits from R&D systems (R&D Systems, Minneapolis, MN, USA). The first step was to prepare the plate coating according to each Certificate of Analysis for each molecule, followed by an overnight incubation at room temperature. After the first incubation, the plates were blocked with 300 μL of reagent diluent. An amount of 100 μL of serum and each standard range were prepared and then each assay protocol was followed step-by-step and at the end, the plates were analyzed with a TECAN SAPRK 10M spectrophotometer (TECAN, Austria GmbH, Grodig, Austria) at 450 nm. The standard range for IL-6 was 9.38–600 pg/mL, for TNF-alpha was 7.8125–1000 pg/mL, and for IFN-beta was 3.91–500 pg/mL.

### 2.3. Statistical Analysis

We defined the following new variable named status of osteolytic lesions after first-line treatment as a dichotomous variable with the following categories: 0 = unchanged osteolytic lesion status before and after first-line treatment, respectively; 1 = changed status (osteolytic lesions present before treatment but absent after).

Demographic and clinical characteristics in all MM samples and stratified by status of osteolytic lesions after treatment were described using the mean (standard deviation) for quantitative variables with normal distribution, median [IQR], IQR = interquartile interval for non-normally distributed data, or absolute (relative) frequency for qualitative nominal variables.

Time-point and longitudinal comparisons of distributions of biomarkers measured at baseline and after first-line treatment, in relation to the changes in osteolytic lesions after first-line treatment were performed by Student’s *t*-test, Mann–Whitney U test, or paired Student-t test or Wilcoxon test.

The direction, magnitude and significance of correlations between modern (IL-6, TNF-alpha, IFN-beta, Free RANKL, RANKL, bone alkaline phosphatase, beta crosslaps), and classical biomarkers (Albumin, Beta2microglobulin, LDH, VSH, Calcium, and hemoglobin) measured at each time point and stratified by status of osteolytic lesions were estimated using non-parametric Spearman’s correlation coefficient.

In order to estimate the longitudinal correlations between modern and classical biomarkers in each of the osteolytic lesion groups, we calculated the change in each biomarker expressed as absolute percent change from baseline using the following formula:

(1) $$Absolute\;percent\;change=\frac{post\_treatment\_value-pre\_treatment\_value}{pre\_treatment\_value}\times 100\%$$

and then we estimated the longitudinal correlations between absolute percent change in each of modern and classical biomarkers using non-parametric Spearman’s correlation coefficient.

All statistical tests were two-sided tests having a significant level a priori set at α = 0.05, and the statistical software used for analyzing sample data was R software version 4.4.0 [14].

## 3. Results

### Baseline Sample Characteristics

Among the 35 patients with MM included in the analysis, seven (20%) of the patients were classified as having changes in bone lesions after first-line treatment (presence of osteolytic lesions before treatment and absence of osteolytic lesions after treatment), while twenty-eight (80%) of the patients did not undergo changes in osteolytic lesions after treatment. The group of patients who displayed unchanged status of osteolytic lesions included twenty-six (92.9%) patients who had osteolytic lesions detected both before and after first-line treatment and two (7.1%) who had no osteolytic lesions before and after first-line treatment. Bisphosphonates were administered as a preventive strategy for the management of bone disease for 26 (74.29%) of MM patients included in this current study. We found no significant association between bisphosphonate administration and the status of osteolytic lesions (Fisher’s exact test, *p* = 1.000, twenty-five (75.0%) in the group with the unchanged status of osteolytic lesions vs. five (71.4%) in the group with the changed status of osteolytic lesions).

The median [IQR] time from MM diagnosis to the post-treatment serum biomarkers collection was 8 [7, 14] months for all samples, the two studied subgroups having a similar distribution of time (median [IQR]: 9 [7, 14] for patients with no changes in status of osteolytic lesions vs. 8 [7, 10] months for patients with changed status of osteolytic lesions, Mann–Whitney U test, *p* = 0.6047).

The age mean (SD) of MM patients was 63.7 (10.9) years, with a significant difference between those with no changes in osteolytic lesions status and patients with changes in osteolytic MM lesions (t(33) = 2.99, *p* = 0.0052). No other significant differences concerning distributions of demographic and clinical characteristics were found between patients with no changes in osteolytic MM lesions and those with changed status of MM osteolytic lesions (Table 1).

When comparing pre-treatment serum biomarkers between patients with and without changes in status of osteolytic lesions, there were no significant differences in values of studied biomarkers between two subgroups (Table 2). For post-treatment serum biomarkers, a significant difference was noticed in Albumin and Calcium, patients with a changed status in osteolytic lesions (absence of lesions after treatment) had higher values of Albumin and Calcium compared to those without changes in status of osteolytic lesions (median [IQR]: 4.2 [4.0, 4.3] vs. 3.5 [3.1, 3.7] for Albumin and 9.2 [8.9, 9.5] vs. 8.7 [8.3, 9.0] for Calcium). When comparing differences between pre- and post-treatment values of biomarkers, patients without changes in status of osteolytic lesions had increased values of Albumin and Hgb and lower values of B2microglobulin and Calcium post-treatment (Table 2). A significant difference in distribution of B2microglobulin values was observed in patients with changes in status of osteolytic lesions (*p* = 0.0469).

The distributions of response to the first-line therapy in the studied groups were as follows: seventeen (60.7%) patients with progressive disease, two (7.1%) patients with stable disease, nine (32.1%) patients with complete remission in the group with unchanged status of osteolytic lesions, two (28.6%) patients with progressive disease, one (14.3%), and four (57.1%) patients with complete remission in the group with changed status of osteolytic lesions. We found no significant association between the status of osteolytic lesions and response to the first-line treatment (Fisher’s exact test, *p* = 0.2713).

**Figure 1:**
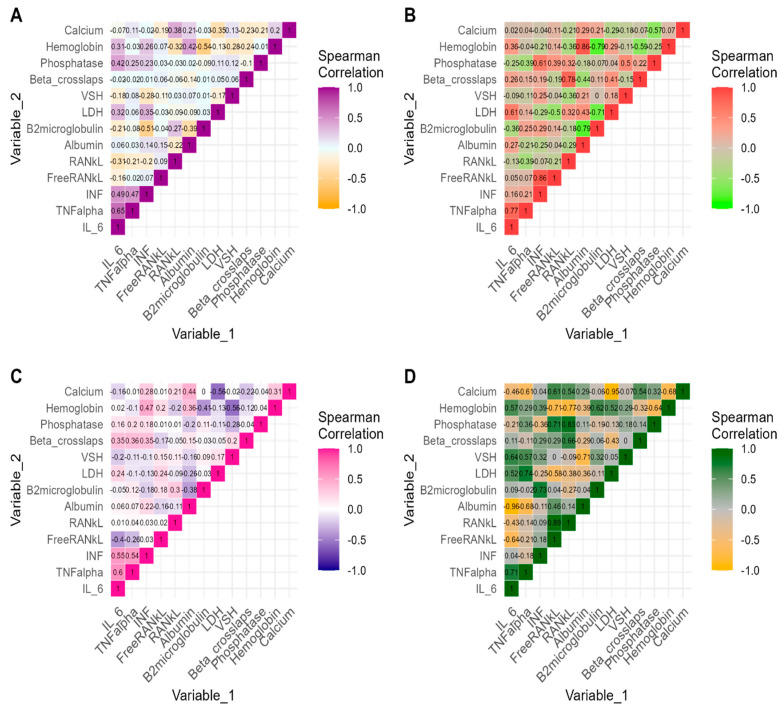
Correlations between modern and classical biomarkers measured at each time point and stratified by status of osteolytic lesions. Note. (**A**) Non-parametric correlations between pre-treatment measures of biomarkers in patients with no changes in status of osteolytic lesions. (**B**) Non-parametric correlations between pre-treatment measures of biomarkers in patients with changes in status of osteolytic lesions. (**C**) Non-parametric correlations between post-treatment measures of biomarkers in patients with no changes in status of osteolytic lesions. (**D**) Non-parametric correlations between post-treatment measures of biomarkers in patients with changes in status of osteolytic lesions. Printed values are the estimates of Spearman’s correlation coefficient. IL-6: Interleukin-6; TNF-α: tumor necrosis factor; IFN: Interferon; FreeRANKL: Free Receptor Activator for Nuclear Factor kappa B Ligand; RANKL: Receptor Activator for Nuclear Factor kappa B Ligand; LDH: Lactate Dehydrogenase; VSH: Erythrocyte Sedimentation Rate. Longitudinal Correlations between modern and classical biomarkers stratified by osteolytic lesion status.

The correlation analysis results between all studied biomarkers are presented in Figure 1. Significant positive strong monotonic correlations were noted between baseline values of ranks and beta crosslaps (ρ = 0.78, *p* = 0.0393), IFN and FreeRank values (ρ = 0.86, *p* = 0.0137), and TNF-alpha and IL-6 (ρ = 0.77, *p* = 0.408) in the patients with changes in status of osteolytic lesions. The baseline values of IL-6 showed a significant positive correlation with IFN (ρ = 0.49, *p* = 0.0087), TNF-alpha (ρ = 0.65, *p* = 0.0002), and phosphatase (ρ = 0.42 *p* = 0.0268) in the patients with no changes in status of osteolytic lesions. In the same group, we also noticed that baseline values of IFN showed a significant negative correlation with B2microglobulin (ρ = −0.51, *p* = 0.0061) and a significant positive correlation with TNF-alpha values (ρ = 0.47, *p* = 0.0121).

After first-line treatment, decreased IL-6 values were significantly correlated with elevated values of Albumin (ρ = −0.96, *p* = 0.0005) in the patients with changes in osteolytic lesions status. Post-treatment values of IFN showed a significant positive correlation with Hemoglobin (ρ = 0.47, *p* = 0.0124), IL-6 (ρ = 0.55, *p* = 0.0026), and TNF-alpha values (ρ = 0.54, *p* = 0.0029).

Correlations between absolute percentage changes from the baseline in biomarkers measured after treatment are displayed in Table 3. At follow-up, changes in β-CTX were mainly positively correlated with Calcium (ρ = 0.96, *p* = 0.0005) and Albumin (ρ = 0.86, *p* = 0.0137), while changes in VSH were negatively correlated with changes in RANKL (ρ = −0.81, *p* = 0.049) in patients with changes in osteolytic lesions. In the same group, we also noticed that changes in IL-6 were negatively correlated with changes in Hemoglobin with a marginal statistical significance (*p* = 0.052).

## 4. Discussion

Multiple myeloma (MM) is a heterogeneous disease with various prognostic factors, and staging systems have been developed to predict the disease outcome. International Staging System (ISS), a risk stratification algorithm, is very useful in establishing survival based on serum Beta2microglobulin and serum Albumin levels.

The prognosis in myeloma is determined by two factors: Beta2microglobulin and serum Albumin. The serum Beta2microglobulin can function as a tumor marker and its level indicates a prognostic factor; it is usually increased (>2.7 mg/L) in 75 percent of patients during diagnosis. A higher value means lower survival [15,16]. Two factors influence the increased prognostic value of serum Beta2microglobulin level: high levels are related to greater tumor burden, which also could be correlated with renal failure that predicts an adverse prognosis. The second factor is the serum’s Albumin level. Lower serum Albumin levels in MM patients are associated with clinical factors like kidney disease or nephrotic syndrome, which reflects the severity of this disease [16,17].

The study conducted by Bataille, Durie, and Grenierj in 2017 evaluated and correlated B2microglobulin with clinical data and the response to chemotherapy of patients with myeloma and demonstrated an increase in the survival rate in patients with a low level of B2microglobulin by up to 52 months compared to an increased level of B2microglobulin, which conferred a much lower survival rate of 26 months. An increased level of Calcium, renal impairment, anemia, and bone lesions (CRAB) are currently accepted as diagnostic criteria for symptomatic (and therefore treatable) myeloma [16].

In patients with MM, anemia is manifested as a Hemoglobin level between 8 and 10 g/dL, while almost 10% have a value below 8 g/dL [6]. Anemia has a negative impact on quality of life and is a predictor of poor survival. The disease progression exacerbates the extent of the anemia, but with treatment, plasmacytosis of bone marrow and hematological and renal parameters were improved [18,19].

Depending on their part in the remodeling process, bone markers are divided into resorption and bone formation markers. Products of osteoclast or collagen degradation form bone resorption markers (β-CTX) while bone alkaline phosphatase-bALP, for example, forms bone formation markers and is produced by osteoclasts. Those can be determined to assess the risk of bone lesions and even fractures, as well as the efficiency of treatment during the therapy [20].

Our study demonstrated that β-CTX levels are higher in MM patients before treatment compared to the post-treatment group. It can be concluded that β-CTX levels are lower in patients without bone damage and increase with the degree of bone damage. Moreover, β-CTX could correlate with the stage of the disease and the extent of neoplastic bone involvement. An elevated level of erythrocyte sedimentation rate is often elevated in MM but could be elevated also in many other hematological malignant tumors like Hodgkin’s disease and chronic lymphocytic leukemia [20,21]. TNF-alpha, a proinflammatory cytokine with a central role in bone pathophysiology, is associated with cell growth, death, and differentiation in multiple myeloma. TNF-alpha can stimulate IL-6 secretion by osteoblasts and stromal cells and accumulated IL-6 stimulates the growth of multiple myeloma cells [22]. TNF-α and IL-6 can be used for therapeutic purpose and, following the conclusion of the studies, it can be observed that an increased level of these markers confers a poor prognosis [23,24].

The molecular examination of cytokines and bone marrow markers in the context of multiple myeloma (MM) involves various techniques for measuring cytokines and chemokines, with ELISA being the most prevalent method for quantifying the protein levels of circulating molecules [12,25,26,27,28]. The correlation between Lactate Dehydrogenase (LDH) and interleukin-6 (IL-6) demonstrated statistical significance in the group with unchanged lytic lesions (*p* = 0.0497), while RANKL exhibited a similar correlation trend (*p* = 0.0003). Conversely, beta crosslaps showed a significant correlation with Albumin (*p* = 0.0137) and Calcium (*p* = 0.0005) when analyzed in relation to the group with changed lytic lesions. To our knowledge, we have not found correlations in the literature between these biomarkers for patients with multiple myeloma.

Our study indicates that elevated levels of β-CTX were observed in myeloma patients exhibiting pre-therapeutic bone disease; thus, extending research to larger patient cohorts could greatly improve the early identification of bone lesions. Furthermore, assessing TNF-alpha alongside IL-6, RANKL, beta crosslaps, IFN, and Free RANKL in a broader patient population may yield valuable insights regarding the condition of bone lesions.

In contrast to traditional markers, classical markers demonstrate enhanced specificity and sensitivity, facilitating more accurate monitoring of the diseases. The expanded application of these markers in multiple myeloma may result in the identification of novel therapeutic options.

One of the disadvantages of modern markers could be related to cost, as their use requires advanced technologies. Another disadvantage might be their more limited availability, as not all medical facilities have access to the necessary technology for determining these markers. The interpretation of results can generally be complex because some of these markers are still under research, and their widespread use is not yet possible.

### Limits of the Study

Due to the SARS-CoV-2 pandemic context, the enrollment of patients in this study was difficult. Probably, the number of enrolled patients would have been higher and the results would certainly have been different. Taking into account that the sample size of the current study was small and the induction regimens highly heterogeneous, this current study should be regarded as exploratory research.

## 5. Conclusions

This current study followed the associations of modern biomarkers with osteolytic lesions status before and after first-line treatment, as well as the cross-sectional and longitudinal correlations between modern and classical biomarkers stratified by osteolytic lesions status.

Upon analyzing the results, it can be concluded that notable differences were identified in post-treatment serum biomarkers, specifically Albumin and Calcium, between patients exhibiting changes in osteolytic lesions and those who did not. Additionally, correlations among various biomarkers, including IL-6, IFN, and TNF-alpha, offered valuable insights into the progression of the disease and the response to treatment. The results obtained from patients with unmodified lytic lesions identified a significant correlation between the biomarkers IL-6, Free RANKL, and IFN-beta with the classical marker LDH. This association highlights the involvement of these markers in promoting bone destruction and the development of osteolytic lesions.

It is important to note that there was no significant relationship found between the administration of bisphosphonates and alterations in osteolytic lesions. Furthermore, only a small percentage of patients (20%) demonstrated changes in osteolytic lesions following first-line treatment, whereas the majority (80%) did not exhibit such changes.

Analyzing the results achieved and identifying the correlations between classic and modern biomarkers can significantly contribute to the advancement of analysis techniques in multiple myeloma.

## Figures and Tables

**Table 1:** Baseline (pre-treatment) patient characteristics stratified by changes in osteolytic lesions after first-line treatment.

	Status of Osteolytic Lesions after First-Line Treatment			
	MM Patients (*n* = 35)	Non-Changed (*n*_1_ = 28)	Changed (*n*_2_ = 7)	*p*-Value
**Demographics characteristics**				
Age at MM diagnosis (years), mean (SD)	63.7(10.9)	66.1 (10.4)	53.7 (6.9)	0.0052 *
Sex, *n* (%)				1.0000
Male	16 (45.7)	13 (46.4)	3 (42.9)	
Female	19 (54.3)	15 (53.6)	4 (57.1)	
**Clinical Characteristics**				
Immune classifications, *n* (%)				
Ig A type	11 (31.4)	10 (35.7)	1 (14.3)	0.3916
Ig G type	19 (54.3)	16 (57.1)	3 (42.9)	0.6772
Ig M type	1 (2.9)	0 (0.0)	1 (14.3)	0.2000
Treatment methods, *n* (%)				0.4341
DVD	1 (2.9)	0 (0.0)	1 (14.3)	
DVMP	4 (11.4)	4 (14.3)	0 (0.0)	
DVTd	5 (14.3)	4 (14.3)	1 (14.3)	
DRd	2 (5.7)	2 (7.1)	0 (0.0)	
VRd	3 (8.6)	2 (7.1)	1 (14.3)	
VCD	12 (34.3)	9 (32.1)	3 (42.9)	
VD	5 (14.3)	5 (17.9)	0 (0.0)	
VEL_DEX	3 (8.6)	2 (7.1)	1 (14.3)	
FLC type (%)				
Kappa	19 (54.3)	14 (50.0)	5 (71.4)	0.4150
Lambda	9 (25.7)	8 (28.6)	1 (14.3)	0.6478

Note. MM = multiple myeloma; SD = sampling standard deviation; FLC = free light chain level; Ig, Immunoglobulin; DVD = Daratumumab Velcade Dexamethazone; DVMP = Daratumumab Velcade Melphalan Dexamethazone; DVTd = Daratumumab Velcade Thalidomida Dexamethazone; DRd = Daratumumab Lenalidomide Dexamethazone; VRd = Velcade Lenalidomide Dexamethazone; VCD = Velcade Cyclophosphamide Dexamethazone; VD = Velcade Dexamethazone. * Significant result at α level = 0.05. Distribution of Biomarkers measured at baseline and after treatment, stratified by changes in osteolytic lesions after treatment.

**Table 2:** Distributions of biomarker values at the baseline and after first-line treatment.

	Pre-Treatment			Post-Treatment				
	Non-Changed Status of Lytic Lesions (*n*_1_ = 28)	Changed Status of Lytic Lesions (*n*_2_ = 7)	*p*-Value	Non-Changed Status of Lytic Lesions (*n*_1_ = 28)	Changed Status of Lytic Lesions (*n*_2_ = 7)	*p*-Value	Non-Changed Status of Lytic Lesions (*n*_1_ = 28)	Changed Status of Lytic Lesions (*n*_2_ = 7)
	Median [IQR] or Mean (SD)	Median [IQR] or Mean (SD)		Median [IQR] or Mean (SD)	Median [IQR] or Mean (SD)		*p*-Value Time Effect	*p*-Value Time Effect
IL-6 (pg/mL)	52.7 [49.1, 97.9]	56.4 [47.6, 136.6]	1.000 ^(a)^	54.1 [48.5, 91.9]	50.5 [48.1, 91.2]	0.9179 ^(a)^	0.1049 ^(c)^	0.2969 ^(c)^
TNF-α (pg/mL)	77.6 [76.5, 141.7]	77.6 [76.4, 98.5]	0.9015 ^(a)^	78.5 [76.7, 149.8]	77.2 [76.8, 93.9]	0.5919 ^(a)^	0.6223 ^(c)^	0.8125 ^(c)^
IFN (pg/mL)	12.1 [7.1, 25.3]	9.4 [6.6, 14.9]	0.5580 ^(a)^	13.9 [6.8, 35.9]	9.7 [7.9, 12.2]	0.4540 ^(a)^	0.7793 ^(c)^	0.1000 ^(c)^
FreeRANKL (pg/mL)	190.2 ^#^ (124.6)	288.2 (179.8)	*0.1059* ^(b)^	219.4 ^##^ (152.6)	274.6 (124.0)	0.3898 ^(b)^	0.2466 ^(d)^	0.8632 ^(d)^
RANKL (pg/mL)	148.1 (133.4)	116.1 ^###^ (104.7)	*0.5031* ^(b)^	154.5 ^#^ (141.6)	169.9 (147.1)	0.8135 ^(b)^	0.1383 ^(d)^	0.1206 ^(d)^
Albumin (g/dL)	3.2 [2.8, 3.7]	3.8 [3.6, 4.4]	0.0549 ^(a)^	3.5 [3.1, 3.7]	4.2 [4.0, 4.3]	0.0029 * ^(a)^	0.0077 * ^(c)^	0.4688 ^(c)^
B2microglobulin (mg/L)	4.9 [4.2, 6.2]	4.6 [4.1, 5.7]	0.5051 ^(a)^	4.4 [3.9, 4.7]	4.5 [3.8, 4.6]	0.4729 ^(a)^	0.0011 * ^(c)^	0.0469 * ^(c)^
Beta crosslaps (ng/mL)	0.6 [0.4, 1.1]	0.5 [0.2, 1.1]	0.5494 ^(a)^	0.4 [0.2, 0.9]	0.4 [0.2, 1.4]	0.3221 ^(a)^	0.3426 ^(c)^	0.4688 ^(c)^
Calcium (mg/dL)	9.0 [8.5, 9.6]	9.3 [9.0, 10.6]	0.1735 ^(a)^	8.7 [8.3, 9.0]	9.2 [8.9, 9.5]	0.0304 * ^(a)^	0.0190 * ^(c)^	0.1563 ^(c)^
Bone alkaline phosphatase (μg/L)	14.1 [10.4, 18.1]	18.9 [11.9, 28.4]	0.3024 ^(a)^	11.6 [9.9, 12.8]	12.6 [11.7, 13.0]	0.1726 ^(a)^	0.2438 ^(c)^	0.4688 ^(c)^
Hgb (g/dL)	10.1 (2.4)	11.5 (3.1)	0.1938 ^(b)^	11.4 (2.3)	12.4 (0.9)	0.1146 ^(b)^	0.0045 * ^(d)^	0.4832 ^(d)^
LDH (U/L)	310.5 [255.0, 395.8]	339.0 [285.5, 387.0]	0.7570 ^(a)^	276.0 [206.8, 401.5]	319.0 [260.0, 358.0]	0.9343 ^(a)^	0.7241 ^(c)^	1.000 ^(c)^
VSH	43.0 [27.0, 56.5]	33.0 [25.5, 54.5]	0.4453 ^(a)^	29.5 [20.5, 36.8]	21.0 [21.0, 26.5]	0.0987 ^(a)^	0.0201 * ^(c)^	0.1056 ^(c)^

Post-treatment = after first-line treatment (in the first month subsequent to the administration of the final first-line treatment course). ^(a)^ Comparisons between subgroups are made with Mann–Whitney U test. ^(b)^ Comparisons between subgroups are made with Student’s *t*-test for independent samples. ^(c)^ Comparisons between pre- and post-treatment were made with paired Student’s *t*-test. ^(d)^ Comparisons between pre- and post-treatment were made with Wilcoxon test; ^#^ *n*_1_ = 25, ^##^ *n*_1_ = 24; ^###^ *n*_1_ = 26. * Significant result at α level = 0.05; IL-6: Interleukin-6; TNF-α: tumor necrosis factor; IFN: Interferon; FreeRANKL: Free Receptor Activator for Nuclear Factor kappa B Ligand; RANKL: Receptor Activator for Nuclear Factor kappa B Ligand; LDH: Lactate Dehydrogenase; VSH: Erythrocyte Sedimentation Rate; Hgb: Hemoglobin Cross-sectional Correlations between modern and classical biomarkers stratified by osteolytic lesions status.

**Table 3:** Relationships between modern and classical biomarkers at follow-up stratified osteolytic lesions status.

Post-Treatment	Non-Changed Status of Lytic Lesions Group (*n*_1_ = 28)		Changed Status of Lytic Lesions Group (*n*_2_ = 7)	
Changes * in Variables	Rho (ρ)	*p*-Value	Rho (ρ)	*p*-Value
IL-6				
Albumin	−0.20	0.4230	0.21	0.6445
Beta2microglobulin	−0.02	0.9405	−0.29	0.5345
LDH	−0.37	0.0497 *	0.14	0.7599
VSH	−0.24	0.2185	0.11	0.2682
Calcium	0.30	0.1188	0.32	0.4821
Hemoglobin	−0.16	0.3094	−0.75	*0.0522*
TNF-alpha				
Albumin	0.11	0.5859	−0.11	0.8192
Beta2microglobulin	−0.22	0.2557	−0.66	0.0938
LDH	−0.33	0.0830	−0.43	0.3374
VSH	0.16	0.4164	0.22	0.6414
Calcium	0.19	0.3274	0.25	0.5887
Hemoglobin	0.18	0.3533	−0.54	0.2152
IFN-beta				
Albumin	−0.19	0.3459	0.39	0.3833
Beta2microglobulin	−0.07	0.7336	−0.11	0.8192
LDH	−0.41	0.0315 *	−0.50	0.2532
VSH	0.02	0.9207	0.11	0.8175
Calcium	0.05	0.8184	0.39	0.3833
Hemoglobin	−0.08	0.6698	−0.18	0.7017
Free RANKL				
Albumin	0.23 ^(a)^	0.2949	0.50	0.2532
Beta2microglobulin	0.19 ^(a)^	0.3878	0.36	0.4316
LDH	0.15 ^(a)^	0.5128	0.61	0.1482
VSH	−0.21 ^(a)^	0.3386	0.0	1.0000
Calcium	−0.38 ^(a)^	0.0772	0.71	*0.0713*
Hemoglobin	−0.29 ^(a)^	0.1892	0.39	0.3833
RANKL				
Albumin	−0.26 ^(b)^	0.2143	0.37 ^(d)^	0.4685
Beta2microglobulin	−0.01 ^(b)^	0.9796	0.49 ^(d)^	0.3287
LDH	−0.67 ^(b)^	0.0003 *	−0.03 ^(d)^	0.9572
VSH	−0.15 ^(b)^	0.4672	−0.81 ^(d)^	0.0499 *
Calcium	0.06 ^(b)^	0.7757	−0.26 ^(d)^	0.6228
Hemoglobin	−0.20 ^(b)^	0.3284	−0.03 ^(d)^	0.9572
Bone alkaline phosphatase				
Albumin	−0.17 ^(c)^	0.3877	0.25	0.5887
Beta2microglobulin	−0.09 ^(c)^	0.6572	0.43	0.3374
LDH	−0.30 ^(c)^	0.1254	−0.39	0.3833
VSH	0.07 ^(c)^	0.7311	−0.49	0.2682
Calcium	0.27 ^(c)^	0.1774	0.00	1.0000
Hemoglobin	0.29 ^(c)^	0.1444	0.29	0.5345
Beta crosslaps				
Albumin	−0.20	0.3018	0.86	0.0137 *
Beta2microglobulin	0.02	0.9229	0.43	0.3374
LDH	−0.17	0.3879	0.21	0.6445
VSH	0.07	0.7313	0.50	0.2482
Calcium	−0.07	0.7210	0.96	0.0005 *
Hemoglobin	0.21	0.2759	0.21	0.6445

* Changes expressed as absolute percent change from baseline (post_treatment value—pre_treament value)/pre_treatment × 100; post-treatment = after first-line treatment (in the first month subsequent to the administration of the final first-line treatment course). ^(a)^ *n*_1_ = 22, ^(b)^ *n*_1_ = 25; ^(c)^ *n*_1_ = 27; ^(d)^ *n*_2_ = 6; IL-6: Interleukin-6; TNF-α:tumor necrosis factor; IFN: Interferon; FreeRANKL: Free Receptor Activator for Nuclear Factor kappa B Ligand; RANKL: Receptor Activator for Nuclear Factor kappa B Ligand; LDH: Lactate Dehydrogenase; VSH: Erythrocyte Sedimentation Rate.

## Data Availability

The datasets used and/or analyzed in this present study are available from the corresponding author upon reasonable request.
